# AAV-mediated human PEDF inhibits tumor growth and metastasis in murine colorectal peritoneal carcinomatosis model

**DOI:** 10.1186/1471-2407-12-129

**Published:** 2012-03-30

**Authors:** Qin Jie Wu, Chang Yang Gong, Shun Tao Luo, Dong Mei Zhang, Shuang Zhang, Hua Shan Shi, Lian Lu, Heng Xiu Yan, Sha Sha He, Dan Dan Li, Li Yang, Xia Zhao, Yu Quan Wei

**Affiliations:** 1 State Key Laboratory of Biotherapy, West China Hospital, West China Medical School, and School of Life Sciences, Sichuan University, Chengdu 610041, P.R. China; 2 Department of Gynecology and Obstetrics, Second West China Hospital, Sichuan University, Chengdu 610041, P.R. China

**Keywords:** AAV, PEDF, Tumor, Antiangiogesis, Gene therapy

## Abstract

**Background:**

Angiogenesis plays an important role in tumor growth and metastasis, therefore antiangiogenic therapy was widely investigated as a promising approach for cancer therapy. Recently, pigment epithelium-derived factor (PEDF) has been shown to be the most potent inhibitor of angiogenesis. Adeno-associated virus (AAV) vectors have been intensively studied due to their wide tropisms, nonpathogenicity, and long-term transgene expression *in vivo*. The objective of this work was to evaluate the ability of AAV-mediated human PEDF (hPEDF) as a potent tumor suppressor and a potential candidate for cancer gene therapy.

**Methods:**

Recombinant AAV_2 _encoding hPEDF (rAAV_2_-hPEDF) was constructed and produced, and then was assigned for *in vitro *and *in vivo *experiments. Conditioned medium from cells infected with rAAV_2_-hPEDF was used for cell proliferation and tube formation tests of human umbilical vein endothelial cells (HUVECs). Subsequently, colorectal peritoneal carcinomatosis (CRPC) mouse model was established and treated with rAAV_2_-hPEDF. Therapeutic efficacy of rAAV_2_-hPEDF were investigated, including tumor growth and metastasis, survival time, microvessel density (MVD) and apoptosis index of tumor tissues, and hPEDF levels in serum and ascites.

**Results:**

rAAV_2_-hPEDF was successfully constructed, and transmission electron microscope (TEM) showed that rAAV_2_-hPEDF particles were non-enveloped icosahedral shape with a diameter of approximately 20 nm. rAAV_2_-hPEDF-infected cells expressed hPEDF protein, and the conditioned medium from infected cells inhibited proliferation and tube-formation of HUVECs *in vitro*. Furthermore, in CRPC mouse model, rAAV_2_-hPEDF significantly suppressed tumor growth and metastasis, and prolonged survival time of treated mice. Immunofluorescence studies indicated that rAAV_2_-hPEDF could inhibit angiogenesis and induce apoptosis in tumor tissues. Besides, hPEDF levels in serum and ascites of rAAV_2_-hPEDF-treated mice were significant higher than those in rAAV_2_-null or normal saline (NS) groups.

**Conclusions:**

Thus, our results suggest that rAAV_2_-hPEDF may be a potential candidate as an antiangiogenic therapy agent.

## Background

Cancer is a major public health problem in the world and causes millions of death each year. More than 1.5 million new cancer cases and 560,000 deaths from cancer are projected to occur in 2010 in the USA [1]. Surgical resection, chemotherapy, and radiotherapy are conventional therapeutic strategies, which were widely used in clinic. Unfortunately, surgical resection is not sufficiently effective for advanced cancer, and chemotherapy and radiotherapy always induce severe side effects [2,3]. Obviously, it is necessary to develop novel therapeutic strategies to optimize available non-surgical approaches. Gene therapy was proposed as a new approach to eradicate malignant cells, which may be beneficial to cancer patients at all stages [4].

Both tumor growth and systemic metastasis are highly dependent on angiogenesis [5]. Tumor less than 1 mm^3 ^could receive necessary nutrients and oxygen through diffusion, however, a tumor is unable to grow above approximate 1 mm^3 ^without neovascularization [6]. Angiogenesis is a complex multistep process consisting of proliferation and migration of endothelial cells, degeneration of basement membrane, and formation of new lumen, which is tightly regulated by both positive and negative factors [7]. Microvascularity of normal tissue is regulated by the proangiogenic and antiangiogenic factors to maintain a quiescent state. Tumor growth and metastasis require neovascularization, therefore, tumors use various mechanisms to shift the balance to an angiogenic inductive environment. In this process, overexpression of proangiogenic factors is accompanied by down regulation of antiangiogenic factors [8]. As angiogenesis is essential for tumor growth and metastasis, antiangiogenesis has been proposed as a therapeutic strategy for cancer treatment [9].

Pigment epithelium-derived factor (PEDF), a 50-kDa secreted glycoprotein, is a member of the serpin superfamily of serine protease inhibitors, which was first identified in a conditioned medium of cultured primary human fetal retinal pigment epithelial cells [10]. To data, it was found that PEDF is widely expressed in human tissues and is involved in many physiological and pathological processes [11]. PEDF is a multifunctional protein and proved to be neurotrophic, neuroprotective, and antiangiogenic. And PEDF has recently been shown to be a more potent inhibitor of angiogenesis than other endogenous angiogenic inhibitors, including angiostatin, endostatin, and thrombospondin-1 [12,13]. Antiangiogenic activity of PEDF was associated with induction of endothelial cell apoptosis via the Fas/FasL death pathway and down-regulation of vascular endothelial growth factor (VEGF) expression. Previous works suggested that PEDF is a potential tumor suppressor [6,14-17].

Although gene therapy is an effective way to treat cancers and many other chronic diseases, the safety and efficacy of gene therapy depends on the development of safe and effective delivery systems [18-23]. The recombinant adeno-associated virus (rAAV) vectors appear to be the promising potential candidate as safe and effective delivery system for gene therapy due to its attractive features [24-27]. rAAV vectors show low immunogenicity and ability to infect both dividing and nondividing cells. Besides, rAAV can maintain long-term undiminished transgene expression *in vivo*. The most important, rAAV vectors are nonpathogenic, which are not associated with any known human diseases. Until now, several different serotypes of AAV have been identified by serological analysis [28-30], among which rAAV serotype 2 vectors (rAAV_2_) are mostly studied and used in animal experiments and clinical trials [31,32]. Most of the previous studies about rAAV-mediated gene transfer of PEDF mainly focused on eye diseases, however, rAAV-mediated gene transfer of PEDF for tumor treatment is rarely reported.

According to the BLAST analysis, the homology between human PEDF and mouse PEDF is very high. Besides, several previous contributions suggested that hPEDF was effective on mouse tumor models. Therefore, in this work, we constructed rAAV_2 _encoding human PEDF (rAAV_2_-hPEDF) and investigated its anti-tumor efficacy in colorectal peritoneal carcinomatosis (CRPC) mouse model. Our results demonstrated that rAAV_2_-hPEDF may be a potent tumor inhibitor and a potential candidate for cancer gene therapy.

## Methods

### Cell lines and animals

CT26 cells (murine colon carcinoma cell line) were purchased from the American Type Culture Collection (ATCC, Rockville, MD) and cultured in RPMI1640 medium (GIBCO, USA) containing 10% fetal bovine serum (FBS, GIBCO, USA) and 1% penicillin/streptomycin. Primary HUVECs were isolated from human umbilical cord veins by a standard procedure [33], and grew in Dulbecco's Modified Eagle's medium (DMEM, GIBCO, USA) supplemented with 20% fetal bovine serum (FBS, GIBCO, USA)and basic fibroblast growth factor (bFGF, 10 ng/ml). HUVECs at passages 2 to 3 were used for experiments. All above cells were maintained at 37°°C in a humidified incubator containing 5% CO_2._

BALB/c mice weighing 20 to 22 g were purchased from the Laboratory Animal Center of Sichuan University and housed at controlled temperature of 20-22°°C, relative humidity of 50-60% and 12 h light-dark cycles. Animals were provided with standard laboratory chow and tap water *ad libitum*. All the animals would be in quarantine for a week before treatment. All animal procedures were performed following the protocol approved by the Institutional Animal Care and Treatment Committee of Sichuan University (Chengdu, P.R. China). All mice were treated humanely throughout the experimental period.

### rAAV vector construction and production

**Figure 1:**
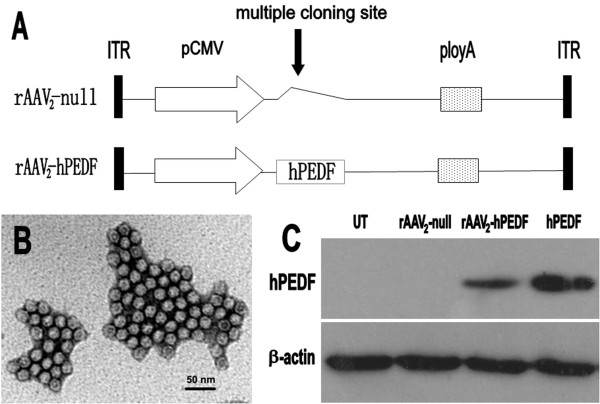
**Construction scheme and characterization of rAAV_2_-hPEDF**. A: Construction scheme of rAAV_2_-hPEDF and control vector rAAV_2_-null; B: TEM image of rAAV_2_-hPEDF particles; C: Expression of hPEDF in rAAV_2_-hPEDF infected CT26 cells.

According to standard cloning techniques, the human PEDF (hPEDF) cDNA was amplified from pBLAST49-hPEDF template (stored by State Key Laboratory of Biotherapy, West China Hospital, Sichuan University, Chengdu, China) using the designed primers (the upstream primer: 5'-GGAATTCATGCAGGCCCTGGTGCTACTC-3'; the downstream primer: 5'-ACGCGTCGACTTAGGGGCCCCTGGGGTC-3'), and then inserted into pAAV_2 _expression vector (provided by Dr. Wu XB, State Key Laboratory for Molecular Virology and Genetic Engineering, Beijing, China) to form the new plasmid, pAAV_2_-hPEDF (Figure 1A). The control plasmid pAAV2-null was constructed in the similar manner (Figure 1A). Packaging and purification of rAAV particles were done as descried previously [34]. The morphological characteristics of rAAV_2_-hPEDF particles were examined by transmission electron microscope (TEM, H-6009IV, Hitachi, Tokyo, Japan). The titers of rAAV_2_-hPEDF and rAAV_2_-null measured by dot blot DNA analysis were 4 × 10^12 ^v.g./mL and 1 × 10^12 ^v.g./mL, respectively. All viruses prepared in this study were stored at 4°°C in PBS before use.

### rAAV_2_-hPEDF infection assay

Approximately 2 × 10^5 ^CT26 cells were plated into 6-well plates in complete medium and allowed to attach for 24 hours. Subsequently, cells were gently washed with PBS, and then rAAV_2_-hPEDF or rAAV_2_-null was applied to cells at MOI (multiplicity of infection) of 10^5 ^v.g. per cell in serum-free medium, with normal saline (NS) as non-infection control. After incubation for 6 hours, complete medium with 10% FBS was added to each well. Then, the conditioned medium was collected after further culture for 72 hours for western blotting analysis and cell proliferation assay.

### Western blotting analysis

Conditioned media from CT26 cells infected with rAAV_2_-hPEDF, rAAV_2_-null or NS were used for Western blotting analysis. Concentrations of protein were determined using a modified Lowry protein assay kit (ThermoScientific), and an equal amount of protein (40 μg) from each sample was loaded on to 10% SDS-PAGE gel followed by transfer to a nitrocellulose membrane sheet, probed for anti-PEDF (1:500, upstates, Lake Placid, USA). The membranes were washed, and blots were developed with horseradish peroxidase (HRP)-conjugated secondary antibodies. The protein bands were detected using an enhanced chemiluminescence (ECL) detection system. (Pierce, Rockford, Illinois, USA).

### Cell proliferation assay

Cell proliferation assay was conducted using 3-(4,5-dimethyl-2-thiazolyl)-2, 5-diphenyl-2H-tetrazolium bromide (methyl thiazolyl tetrazolium, MTT) method to determine the effect of hPEDF derived from rAAV_2_-hPEDF infected cells on HUVECs. HUVECs were plated at a density of 1 × 10^4 ^cells per well in 100 μL of medium in 96-well plates and grown for 48 hours. The conditioned media from CT26 cells infected with rAAV_2_-hPEDF, rAAV_2_-null, or NS were collected as described above, and each supernatant was two-fold serially diluted and added to each well in triplicate. After 48 hours of incubation, viability of cells was measured using MTT method. The mean percentage of cell survival relative to that of untreated cells was estimated from data from three individual experiments, and all data were expressed as the mean value ± standard deviation (S.D.).

### Endothelial cell capillary-like tube formation assay

Tube formation assay was conducted as previously described [35]. Briefly, prechilled Matrigel was pipetted into prechilled 24-well plates and polymerized at 37°°C for 30 minutes. Then, HUVECs suspended in complete medium were seeded onto the Matrigel, and they were treated with conditioned medium form cells infected with NS, rAAV_2_-null, or rAAV_2_-hPEDF, respectively. After 6 hours of incubation, the endothelial cells were photographed with a digital camera attached to an inverted microscope. Three independent experiments were performed.

### Animal model and treat plan

CRPC mouse model was established and subsequently treated. Briefly, the mice were intraperitoneally injected with 200 μl of cell suspension containing 5 × 10^5 ^CT26 cells. Tumors were allowed to grow for 3 days. Tumor-bearing mice were randomly assigned to one of the following groups (n = 12 mice per group): NS, rAAV_2_-null (2 × 10^10 ^v.g. per mouse), and rAAV_2_-hPEDF (2 × 10^10 ^v.g. per mouse).

Treatments were administered through intraperitoneal injection with a single dose of 200 μl above mentioned viruses. For tumor growth inhibition study (6 mice per group), the number and weight of tumor nodes in each group were measured on day 18 after tumor cell inoculation. After day 18, NS and rAAV_2_-null-treated mice began to die. To further study the therapeutic effect against colorectal cancer, the survival time of mice treated with the protocols described above was observed (6 mice per group).

### Detection of microvessel density (MVD)

The antiangiogenesis of rAAV_2_-hPEDF was determined by immunofluorescent analysis of neovascularization in tumor tissue as described [36]. Briefly, frozen sections of tumors were fixed in acetone, washed with PBS, stained with rat anti-mouse CD31 (platelet endothelial cell adhesion molecule-1) polyclonal antibody (1:50; BD Pharmingen™, USA), washed twice with PBS, and followed by incubation with a Rhodamine-conjugated second antibody (Abcam, USA). MVD was determined by counting the number of microvessels per high-power field in the sections with a fluorescence microscopy as described.

### Quantitative assessment of apoptosis

Tumor sections were prepared as described above. Terminal deoxynucleotidyl transferase-mediated nick-end labeling (TUNEL) staining was done using an *in situ *cell death detection kit (DeadEnd™ Fluorometric TUNEL System, Promega, Madison, USA) following the manufacturer's protocol. It is based on the enzymatic addition of digoxigenin-nucleotide to the nicked DNA by the recombinant terminal deoxynucleotidyl transferase (rTdT) [37]. In the tissue sections, four equal-sized fields were randomly chosen and analyzed. The apoptotic index was calculated as a ratio of the apoptotic cell number to the total tumor cell number in each high-power field.

### Detection of hPEDF concentrations in serum and ascites

Quantitation of hPEDF expression in serum and ascites was determined by enzyme-linked immunosorbnent assay (ELISA). A commercial PEDF ELISA kit (ADL, Biotech. Dev. Co., USA) was used following the manufacturer's instructions. Briefly, pre-diluted serum or ascites were added to wells of the pre-coated ELISA plate and incubated for one hour. After washing, HRP-conjugated secondary antibody was added. Antibody binding was detected with substrate A and B. After the reaction was stopped with H_2_SO_4_, the absorbance at 450 nm was measured by an ELISA reader (Bio-Rad 680, USA).

### Toxicity evaluation

To investigate potential toxicity or side effects of rAAV_2_-hPEDF, mice were observed continuously during the treatment process, including the general conditions (the activity, energy, hair, feces, behavior pattern, and other clinical signs), body weight, and mortality. For histopathological studies, samples were obtained from major organs including heart, liver, spleen, lungs, and kidneys. All the samples were fixed in 10% neutral-buffered formalin, and embedded in paraffin. Then, the tissues were sectioned and stained with hematoxylin and eosin (H&E).

### Statistical analysis

The statistic analysis was carried out using SPSS 15.0 software (Chicago, IL, USA). Comparison of numbers of tumor nodes was performed using one-way analysis of variance (ANOVA). Survival curves were generated based on the Kaplan-Meier method and statistical significance was determined by Mann-Whitney U-tests. A *P *value < 0.05 on a 2-tailed test was considered statistically significant.

## Results

### Expression of hPEDF in colonic cancer cells after rAAV-mediated gene transfer *in vitro*

rAAV_2_-hPEDF was successfully constructed (Figure 1A). TEM was used to determine the morphological characteristics of rAAV_2_-hPEDF particles. As presented in Figure 1B, rAAV_2_-hPEDF particles were non-enveloped icosahedral shape with a diameter of approximately 20 nm. To determine whether hPEDF could be secreted by rAAV_2_-hPEDF-infected cells, we analyzed the conditioned medium from CT26 cells infected *in vitro *with either rAAV_2_-hPEDF or control vector rAAV_2_-null by Western blotting analysis using a monoclonal antibody against hPEDF. As shown in Figure 1C, in the conditioned medium from rAAV_2_-hPEDF-infected cells, only one band with molecular weight of 50 kDa was observed, which coincided with the lane loaded with hPEDF protein. There was no hPEDF expression in the conditioned medium from the cells infected with control vector rAAV_2_-null or NS control. This result suggested that rAAV_2_-hPEDF could transfer hPEDF gene into cultured cells and produce secretory protein.

### Biological activity of hPEDF produced by rAAV_2_-hPEDF *in vitro*

To evaluate the bioactivity of secreted hPEDF from CT26 cells infected with rAAV_2_-hPEDF, HUVEC proliferation assay and endothelial cell capillary-like tube formation assay were conducted.

**Figure 2:**
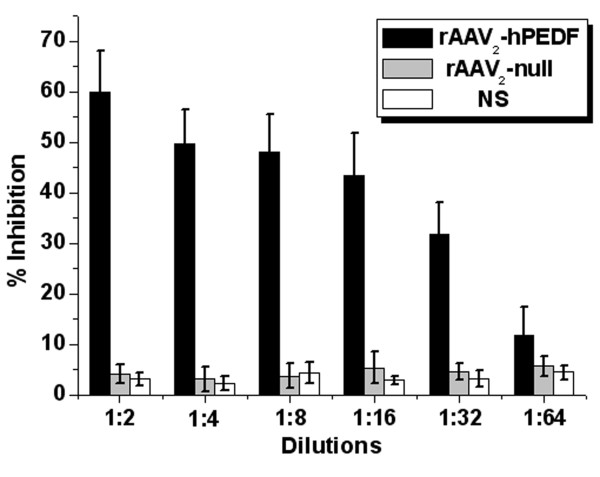
**Inhibitory effect of rAAV_2_-hPEDF on proliferation of HUVECs *in vitro***. Exponentially growing HUVECs were exposed to the conditioned medium from rAAV_2_-hPEDF, rAAV_2_-null, or NS infected CT26 cells for 72 hours. Percent inhibition was calculated by the MTT method. Columns, mean of three independent experiments; error bars, SD.

For *in vitro *HUVEC proliferation assay, HUVECs were exposed to the conditioned media from CT26 cells infected with rAAV_2_-hPEDF, rAAV_2_-null, or NS, respectively. After 48 hours of incubation, viability of HUVECs was measured using MTT method. We found that incubation with the conditioned medium from CT26 cells infected with rAAV_2_-hPEDF significantly inhibited HUVEC proliferation compared with the conditioned media from cells infected with rAAV_2_-null or NS. According to Figure 2, the conditioned medium from cells infected with rAAV_2_-hPEDF inhibited HUVEC proliferation by 59.9 ± 8.1% at a 1:2 dilution concentration, and the inhibitory rate decrease with the increase of dilution concentration showing a dose-dependent effect. In contrast, the conditioned medium from rAAV_2_-null had no inhibitory effect on the proliferation of HUVECs.

**Figure 3:**
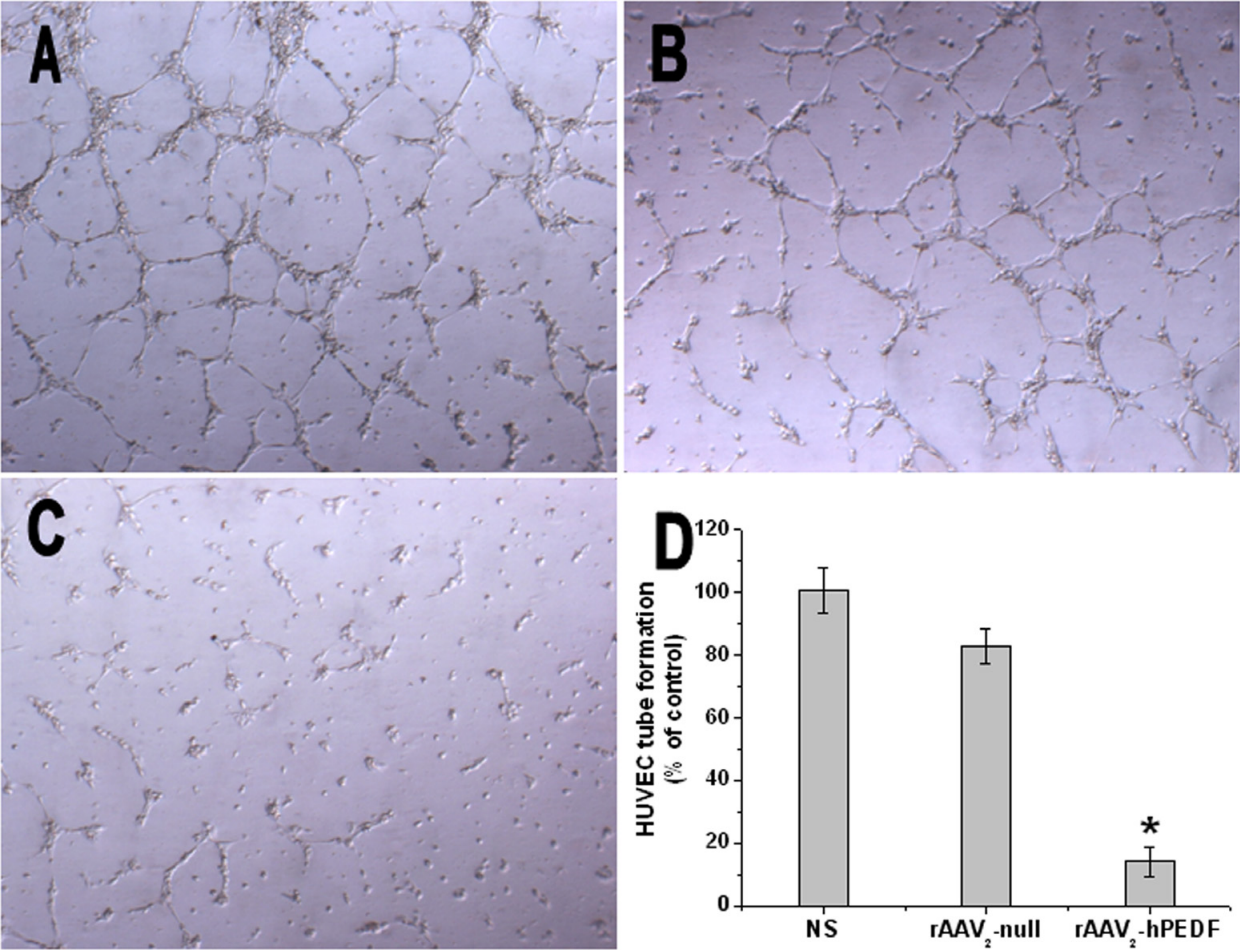
**rAAV_2_-hPEDF inhibited tube formation of HUVECs**. Representative photographs of HUVECs treated with the conditioned medium from NS (A), rAAV_2_-null (B), or rAAV_2_-hPEDF (C) infected CT26 cells, and inhibition ratio of tube formation (D). Columns, mean of three independent experiments; error bars, SD.

Although angiogenesis is a complex process of several kinds of cells, tube formation of endothelial cells is one of the key steps of angiogenesis. Two-dimensional Matrigel assay was used to examine the potential effects of conditioned media from cells infected with rAAV_2_-hPEDF, rAAV_2_-null, or NS on the capillary-like structure formation of endothelial cells, respectively. As shown in Figure 3, HUVECs seeded on the surface of Matrigel formed capillary-like structures in conditioned medium from NS or rAAV_2_-null group within 6 hours. However, treatment with the conditioned medium from cells infected with rAAV_2_-hPEDF strongly reduced the tube formation of endothelial cells (Figure 3D).

These results indicated that the hPEDF secreted by infected cells was highly bioactive and could block angiogenesis *in vitro *by inhibiting the proliferation and reducing the formation of tube-like structure of endothelial cells.

### Inhibition of tumor growth and metastasis with rAAV_2_-hPEDF

**Figure 4:**
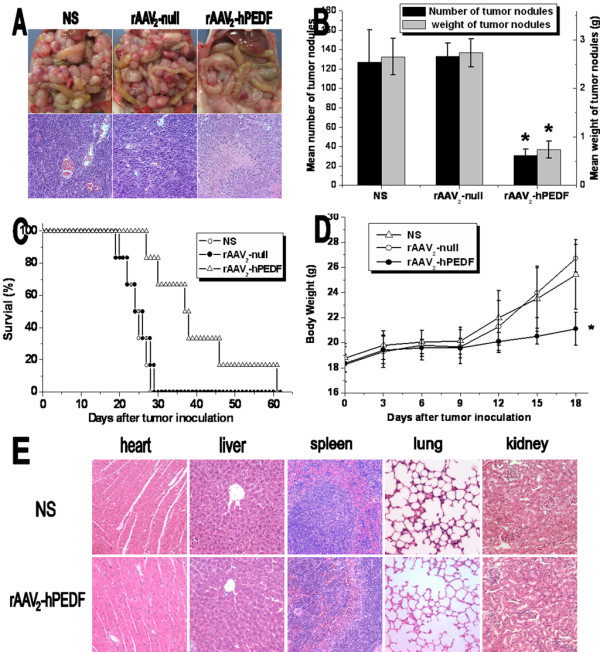
**rAAV_2_-hPEDF inhibited growth and metastasis in CRPC model, and prolonged survival time of treated mice**. A: Representative photographs and H&E staining of abdominal tumors in NS (a), rAAV_2_-null (b), and rAAV_2_-hPEDF (c) groups; B: Mean number and weight of tumor nodules in each group; C: Survival curves of mice in each group; D: Body weight of mice in each group; E: Photographs of major organs after intraperitoneal administration of rAAV_2_-hPEDF and NS (×400).

To investigate the therapeutic effect of rAAV_2_-hPEDF, forty-eight CRPC mice were divided into three groups and treated with NS, rAAV_2_-null, or rAAV_2_-hPEDF, respectively. On day 18 after cell inoculation, mean number and weight of tumor nodes in rAAV_2_-hPEDF-treated group were dramatically decreased compared with those in rAAV_2_-null (*P *< 0.001 and *P *< 0.001, respectively) or NS group (*P *< 0.001 and *P *< 0.001, respectively) (Figure 4A and 4B). Besides, the size of tumor nodes in rAAV_2_-hPEDF-treated group was significantly smaller in comparison with control groups (Figure 4A). According to H&E staining of tumor in each group, less vessels and remarkable necrosis areas were observed in tumor tissues from rAAV_2_-hPEDF group (Figure 4A). These findings indicated rAAV_2_-hPEDF not only inhibits growth of implanted tumor, but also impairs tumor metastasis. Furthermore, there was a substantial increase in the life span of the rAAV_2_-hPEDF-treated mice, and the median survival of rAAV_2_-hPEDF group was 37 days versus 24 days or 25 days in rAAV_2_-null or NS group, respectively (Figure 4C). Besides, due to the inhibition of tumor growth and metastasis, body weight of mice in rAAV_2_-hPEDF-treated group increased slower compared with that in NS or rAAV_2_-null groups (Figure 4D). Mice were investigated in particular for potential side effects attributable to rAAV_2_-hPEDF therapy. No severe toxic effects were observed in gross measures, such as severe weight loss, changes in behavior, and feeding. Moreover, no histopathological changes were found in major organs including heart, liver, spleen, lungs, and kidneys (Figure 4E).

### Inhibition of tumor angiogenesis

**Figure 5:**
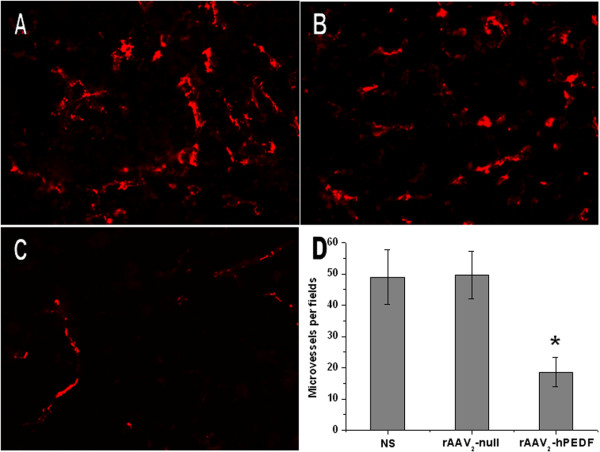
**CD31 immunofluorescent staining of tumor nodules**. Representative CD31 immunofluorescent images of NS (A), rAAV_2_-null (B), and rAAV_2_-hPEDF (C) group, and mean MVD in each group.

To better understand the mechanism by which treatment with rAAV_2_-hPEDF inhibited the tumor growth and metastasis, the MVD of each group was measured. Sections of tumor nodes from mice in all three treatment groups were stained for CD31 immunofluorescence to determine the MVD as a measure of tumor angiogenesis (Figure 5). As shown in Figure 5A to 5C, significant fewer immunoreactive microvessels were observed in tumor tissue from mice treated with rAAV_2_-hPEDF. According to Figure 5D, MVD of tumor tissues from rAAV_2_-hPEDF-treated mice (18.6 ± 4.7) was significant lower compared with control vessel rAAV_2_-null (49.6 ± 7.7, *p *< 0.001) or NS group (49.0 ± 8.7, *p *< 0.001).

### Induction of tumor apoptosis

**Figure 6:**
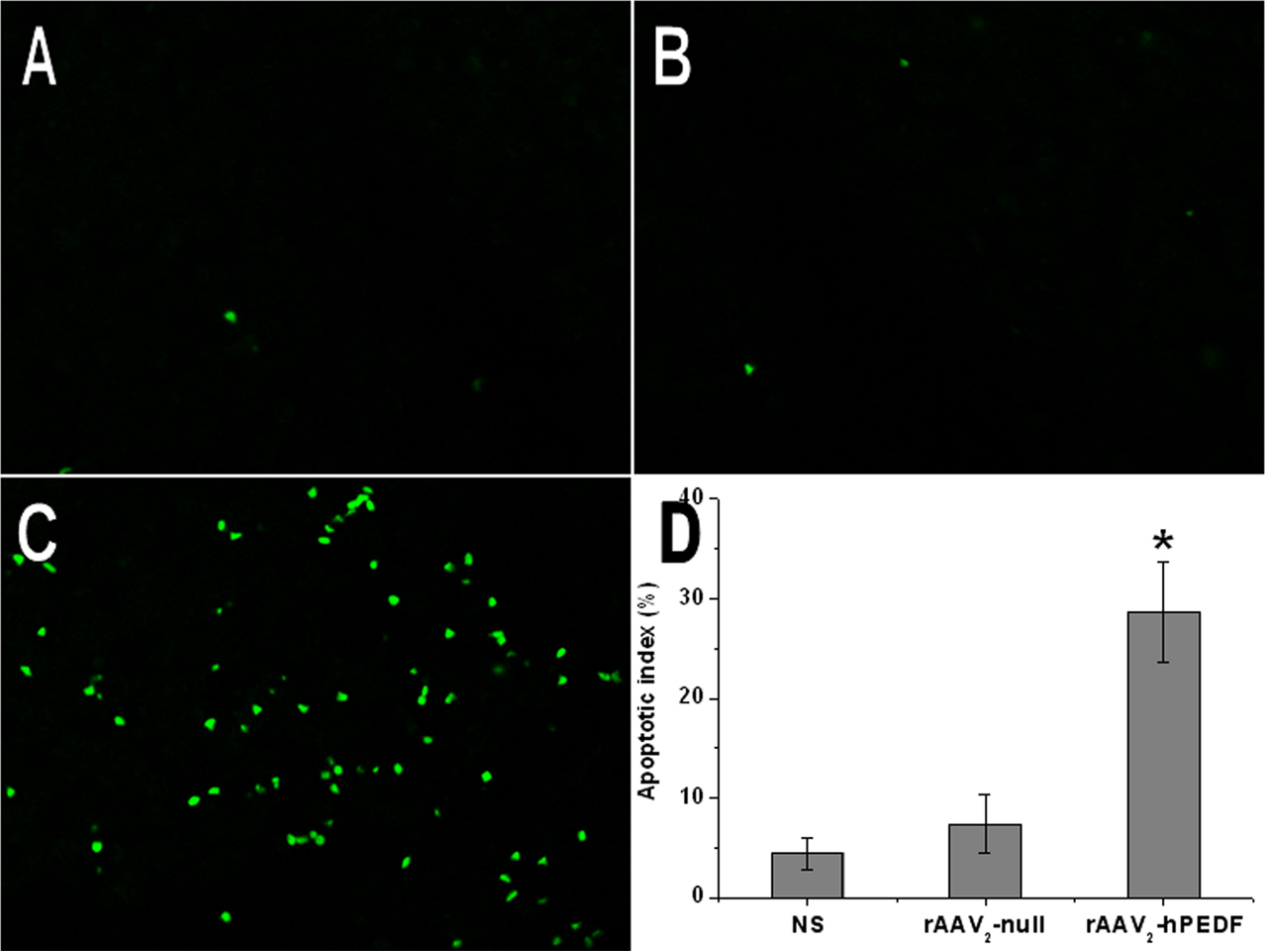
**TUNEL immunofluorescent staining of tumor nodules**. Representative TUNEL immunofluorescent images of NS (A), rAAV_2_-null (B), and rAAV_2_-hPEDF (C) group, and mean apoptotic index in each group.

To explore the role of rAAV_2_-hPEDF on apoptosis of tumor cells, tumor resections were subjected to immunofluorescent TUNEL staining assays for respective determination of apoptotic index. As shown in Figure 6A to 6C, within a similar high-power field, more apoptotic cells (with green nuclei) in tumor tissues were observed in rAAV_2_-hPEDF-treated mice compared with those in rAAV_2_-null or NS group. The apoptosis index was significantly higher in rAAV_2_-hPEDF group (28.6% ± 5.0%) than in rAAV_2_-null (7.4% ± 2.9%, *p *< 0.001) or NS group (4.4% ± 1.5%, *p *< 0.001), respectively (Figure 6D).

### hPEDF concentrations in serum and ascites

**Figure 7:**
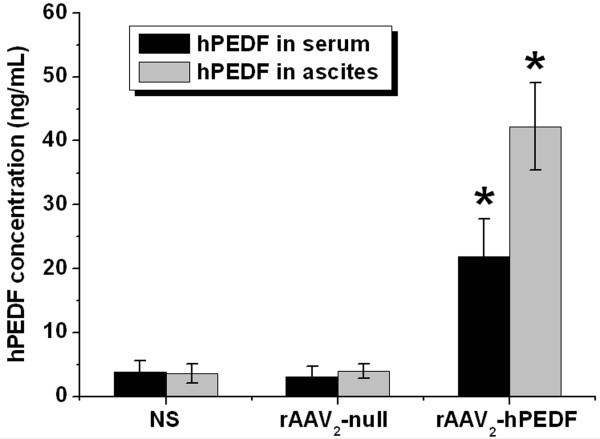
**hPEDF concentrations in serum and ascites at day 18 after tumor inoculation**. hPEDF levels in serum and ascites of rAAV_2_-hPEDF-treated mice were significant higher than those in rAAV_2_-null (*P *< 0.001) or NS (*P *< 0.001) groups.

To evaluate whether the suppression of tumor growth and metastasis and increase of life span in rAAV_2_-hPEDF-treated group is associated with the hPEDF expression, hPEDF concentrations in serum and ascites were measured in each treatment groups. Data in Figure 7 show the hPEDF levels in serum and ascites on day 18 after tumor cell inoculation. hPEDF levels in serum (21.87 ± 5.94 ng/mL) or ascites (42.23 ± 6.80 ng/mL) in rAAV_2_-hPEDF-treated group were significantly higher than those in rAAV_2_-null (3.03 ± 1.61 ng/mL in serum, *P *< 0.001; 3.95 ± 1.09 ng/mL in ascites, *P *< 0.001) or NS (3.83 ± 1.76 ng/mL in serum, *P *< 0.001; 3.57 ± 1.47 ng/mL in ascites, *P *< 0.001) group, respectively. Furthermore, in rAAV_2_-hPEDF-treated group, hPEDF level in ascites was significantly higher than that in serum (*P *< 0.001). The results indicated that intraperitoneal injection of rAAV_2_-hPEDF produced high level secretory hPEDF protein in mice, which inhibited the growth and metastasis of tumor.

## Discussion

Colorectal carcinoma is one of the most frequent cancers, and is the third leading cause of cancer-associated deaths worldwide [7]. Besides liver metastasis and regional lymph nodes metastasis, peritoneal carcinomatosis of colon cancer occurs in 12-20% of patients whose disease recurs [38,39]. However, therapeutic effects of conventional treatments, such as surgical resection, chemotherapy and radiotherapy, are not very encouraging. Therefore, unremitting efforts were focused on developing novel therapeutic strategies. Data presented in this study showed that AAV-mediated delivery of hPEDF could prevent tumor growth and metastasis and prolong the survival time in murine CRPC model (Figure 4A to 4D).

Angiogenesis is essential to maintain tumor growth and metastasis, therefore antiangiogenic therapy is a feasible approach for cancer therapy. In recent years, a variety of angiogenesis inhibitors have been discovered, among which PEDF is the most promising one. Based on previous studies, down-regulation of PEDF e was very prevalent in a range of tumors [11]. There are two major pathways by which PEDF exerts its antitumor effects: antiangiogenesis and apoptosis-mediated tumor suppression. In this study, we examined biological activity of hPEDF produced by rAAV_2_-hPEDF *in vitro *and *in vivo*. In HUVEC proliferation assay, the conditioned medium from cells infected with rAAV_2_-hPEDF inhibited HUVEC proliferation by 59.9 ± 8.1% at a 1:2 dilution concentration (Figure 2). In HUVEC tube formation assay, treatment with the conditioned medium from cells infected with rAAV_2_-hPEDF dramatically reduced the tube formation of HUVECs by 85.8% ± 4.7% (Figure 3). Furthermore, sections of tumors stained for CD31 immunofluorescence showed that the MVD of tumors in rAAV_2_-hPEDF-treated mice was reduced by 62.1 ± 9.6% compared with control group. In immunofluorescent TUNEL staining assay, the apoptosis index in rAAV_2_-hPEDF group increased by 6.5 times compared with NS group (Figure 6). Therefore, our *in vitro *and *in vivo *results suggested that suppression of tumor growth and metastasis by rAAV_2_-hPEDF was associated with the decreased MVD and induction of apoptosis in tumors.

Antiangiogenic therapy requires constant therapeutic levels of antiangiogenic factors *in vivo *to achieve its therapeutic effect, therefore recombinant proteins are limited for widespread clinical use [40]. It is because high therapeutic doses of recombinant proteins are needed, but resultant yield rates of recombinant proteins may be low due to denaturizing in purification process. Furthermore, owing to the short half-life of protein *in vivo*, maintaining therapeutically effective serum levels needs a frequent dosing regimen and high doses of expensive purified recombinant proteins. Thus, efforts should be made to develop effective and wide-applied strategy, and one potential solution for this is gene therapy. The key of successful gene therapy is gene delivery system. AAV, as one of the most promising viral vectors for human gene therapy, has many advantages compared to other viral vectors, such as non-pathogenicity, low immunogenicity, and long-term undiminished transgene expression *in vivo*. Since the first infectious clone of AAV_2 _was established in 1982, AAV_2 _vectors have gained increasing attention in gene therapy applications [41,42]. AAV_2 _have been extensively investigated in preclinical studies for many diseases, including hemophilia, rheumatoid arthritis, cystic fibrosis, and etc [42,43]. However, further studies suggested that most humans exposed to AAV_2 _would develop neutralizing antibodies to the vector, which hindered its transduction efficiency [44,45]. Although the application of AAV_2 _hampers clinical potential of this approach, the use of well-studied AAV_2 _will provide preliminary proof of efficacy which would warrant future studies using vectors derived from other serotypes. In this study, we successfully constructed and produced rAAV_2_-hPEDF, and the titer of rAAV_2_-hPEDF was 4 × 10^12 ^v.g./mL. Besides, the morphological characteristics of rAAV_2_-hPEDF were detected by TEM, which exhibited that rAAV_2_-hPEDF particles were non-enveloped icosahedral shape with a diameter of approximately 20 nm (Figure 1B). A single intraperitoneal administration of rAAV_2_-hPEDF resulted in suppression of tumor growth and metastasis in the CRPC mouse model with maximum inhibition of 75.85% (Figure 4B). Besides, our results indicated that a single intraperitoneal administration of rAAV_2_-hPEDF could increase hPEDF levels in serum and ascites for a long period (Figure 7), which could continue to inhibit angiogenesis in tumor. During the treatment process, no severe toxic effects were observed, and no histopathological changes were found in major organs (Figure 4E). All these data confirmed that AAV is a safe and efficient gene delivery vector.

## Conclusions

In conclusion, our studies demonstrated that rAAV_2_-hPEDF could infect cells and produce secretory hPEDF protein, which was proved to be functional on inhibiting proliferation and tube-formation of HUVECs *in vitro*. Moreover, rAAV_2_-hPEDF significantly suppressed tumor growth and metastasis and prolonged survival time of treated mice in CRPC model. rAAV_2_-hPEDF could increase hPEDF levels in serum and ascites, inhibit angiogenesis, and induce apoptosis in tumor tissues. Therefore, our results indicated that rAAV_2_-hPEDF may be a potential candidate as an antiangiogenesis therapy agent for cancer gene therapy.

## Abbreviations

ANOVA: One-way analysis of variance; bFGF: Basic fibroblast growth factor; CRPC: Colorectal peritoneal carcinomatosis; DMEM: Dulbecco's Modified Eagle's medium; ECL: Enhanced chemiluminescence; ELISA: Enzyme-linked immunosorbnent assay; FBS: Fetal bovine serum; hPEDF: Human pigment epithelium-derived factor; HUVECs: Human umbilical vein endothelial cells; H&E: Hematoxylin and eosin; IL-8: Interleukin-8; MTT: Methyl thiazolyl tetrazolium; MVD: Microvessel density; NS: Normal saline; PEDF: Pigment epithelium-derived factor; rAAV: Recombinant adeno-associated virus; rAAV_2_: Recombinant adeno-associated virus serotype 2 vectors; rAAV_2_-hPEDF: Recombinant adeno-associated virus serotype 2 vectors encoding human pigment epithelium-derived factor; S.D.: Standard deviation; TUNEL: Terminal deoxynucleotidyl transferase-mediated nick-end labeling; VEGF: Vascular endothelial growth factor.

## Competing interests

The authors report no competing interests. The authors alone are responsible for the content and writing of the paper.

## Authors' contributions

LY, YQW, and QJW designed the experiments. And the research funds were supported by LY and YQW. QJW and CYG carried out experiments, analyzed the data, and wrote the manuscript; LY and XZ corrected the manuscript. STL participated in the construction of pAAV_2_-hPEDF. HSS participated in packaging and purification of rAAV particles. DMZ participated in Western blotting analysis. SZ participated in HUVECs tube formation test. LL, HXY, SSH, and DDL participated in the animal experiments and immunofluorescence assay. All authors approved and read the final manuscript.

## Pre-publication history

The pre-publication history for this paper can be accessed here:

http://www.biomedcentral.com/1471-2407/12/129/prepub
